# Wnt pathway-related three-mRNA clinical outcome signature in bladder urothelial carcinoma: computational biology and experimental analyses

**DOI:** 10.1186/s12967-021-03061-4

**Published:** 2021-09-27

**Authors:** Siqing Sun, Yutao Wang, Jianfeng Wang, Jianbin Bi

**Affiliations:** Department of Urology, China Medical University, The First Hospital of China Medical University, Shenyang, 110001 Liaoning China

**Keywords:** Wnt pathway, EMT, Computational biology, PPP2CB, Risk signature

## Abstract

**Background:**

The Wnt signaling pathway is core to the growth of bladder tumors. Epithelial-to-mesenchymal transition (EMT) is significant for bladder tumor metastasis. Nevertheless, the relationship between the Wnt signaling pathway, outcomes of bladder cancer (BLCA), and the specific mechanisms driving immune infiltration have not been studied.

**Methods:**

We obtained Wnt pathway-related gene mRNA and clinicopathological data from the Cancer Genome Atlas (TCGA). We obtained 34 genes that were greatly correlated with outcome using univariate Cox regression analysis and conducted a completely randomized data t-test to perform clinical staging. According to the single-sample gene set enrichment analysis (ssGSEA), the weighted correlation network analysis (WGCNA) was applied to identify relevant biological functions. Various subtypes were identified using consensus cluster analysis. Univariate Cox regression and least absolute shrinkage sum selection operator–Cox regression algorithm analysis were conducted on TCGA and Gene Expression Omnibus data to identify risk characteristics. The Kaplan–Meier method and receiver running feature curves were adopted to calculate overall survival. Single-sample gene set enrichment analysis (ssGSEA) was adopted for the assessment of the degree of immune infiltration. Then, we demonstrated the relationship between PPP2CB and EMT function in two cell lines.

**Results:**

Thirty-four Wnt signaling pathway-related genes were risk factors for BLCA outcome, and their expression levels differed by clinical stage. The co-expression of WGCNA showed the relationship between the Wnt signaling pathway and biological functions and was closely associated with EMT. We divided BLCA patients into two subtypes using consensus clustering. Survival curves and clinical analysis showed that the Wnt pathway enriched group had worse outcomes. The Wnt signature showed the significance of the outcome for MAPK10, PPP2CB, and RAC3. Based on these genes, the degree of immune infiltration was evaluated. Cell function experiments suggested that PPP2CB drives the proliferation and migration of BLCA cells.

**Conclusion:**

We found that Wnt signaling pathway-related genes can be used as prognostic risk factors for BLCA, and the Wnt signaling pathway is a cancer-promoting signaling pathway associated with EMT. We identified three critical genes: MAPK10, RAC3, and PPP2CB. The genes in these three Wnt signaling pathways are associated with tumor cell EMT and immune cell infiltration. The most important finding was that these three genes were independent prognostic factors for BLCA.

**Supplementary Information:**

The online version contains supplementary material available at 10.1186/s12967-021-03061-4.

## Introduction

Bladder cancer (BLCA) is the tenth most usual cancer and ranks sixth among males. It is associated with high incidence and mortality rates. There are approximately 549,393 new cases and 199,922 deaths in the world annually [1]. The Wnt signaling pathway is a relatively classic signaling pathway related to cell differentiation, migration and cell polarity. The Wnt signaling pathway is fallen into three sub-pathways: the classic β-catenin-dependent path, the non-canonical Wnt/calcium path and the non-canonical planar cell polarity path. Abnormal Wnt signaling is related to some cancers, the most remarkable ones being colorectal, breast, lung, breast, oral, and cervical cancer and hematopoietic malignancies [2]. Most studies of the Wnt signaling pathway in BLCA only focused on the Wnt pathway as a downstream signaling pathway under the regulation of upstream molecules or medications to regulate the proliferation and differentiation of BLCA cells. These studies did not mention the influence of the Wnt signaling pathway on BLCA outcomes. Therefore, the current research determined the relationship between the Wnt signaling pathway and BLCA outcome and identified independent prognostic factors and the effects of Wnt pathway-related genes on BLCA outcome by using RNA-seq data downloaded from The Cancer Genome Atlas (TCGA). In addition, a prognostic model was set up on basis of recognized genes, the applicability and value of the model in BLCA were assessed; the generalizability of the model was studied using external verification using the Gene Expression Omnibus (GEO).

## Materials and approaches

### Achievement of BLCA expression profiles from TCGA datasets

UCSC Xena provided RNA-seq data (level 3) and clinical data of BLCA samples. The Genome Tissue Expression (GTEx) [3] tool was adopted to collect expression levels of genes discussed in normal tissues. Then, the “sva” package of R software [4] was used to normalize RNA expression profiles and remove batch effects. GSE13507 [5] was also obtained from GEO, the platform for which was GPL6102. The GSE13507 cohort contained 165 primary BLCA samples, 58 normal-looking bladder mucosa samples surrounding cancer, 23 recurrent non-muscle invasive tumor tissues, and ten normal bladder mucosae.

### Analysis of clinical phenotype of the Wnt signaling path

We downloaded the complete list of Wnt path genes from KEGG [6]. The list included several Wnt pathway-related genes (Additional file 1: Table S1). To identify the Wnt signaling pathway genes associated with BLCA outcome, we downloaded the relevant genes of the Wnt signaling pathway and performed univariate Cox regression analysis applying TCGA (P < 0.05). To characterize Wnt-related gene expression patterns in BLCA, RNA sequencing data in TCGA samples were analyzed. Subsequently, based on the median value of Wnt pathway activation in the sample, all patients were fallen into two groups (activated and inhibited), and KEGG was used for difference analysis to create a volcano map.

### ssGSEA and WGCNA analysis for the analysis of Wnt-related biological functions

Based on TCGA BLAD samples, single-sample gene set enrichment analysis (ssGSEA) [7] analysis was employed for the evaluation of 6935 Gene Oncology (GO) [8] biological functions of each sample. Five Wnt pathways were recognized applying weight gene co-expression network analysis (WGCNA) [9] clinical phenotype, and then module assignment analysis was performed based on WGCNA. We obtained the correlation diagrams for the ssGSEA scores of GO functions and the Wnt pathway. We found epithelial-mesenchymal transition (EMT)-related biological functions were the GO terms most associated with the Wnt pathway. Use WGCNA (version: 1.61) for co-expression network analysis. First, we select the soft threshold for building the network. The soft threshold gives the adjacency matrix a continuous value between 0 and 1, so that the established network meets the power law distribution and is near the actual state of the biological network. Second, we use the blockwise modules function to build a ladderless network, and then identify the gene co-expression modules that cluster genes with similar expression patterns by performing modulus assignment analysis. The definition of module is made by cutting the clustering tree into branches and assigning them different colors with the dynamic tree cutting algorithm. Then the Eigengene modulus (ME) of every modulus is calculated. ME represents the expression level of each module. Then, the association between the ME and the clinical characteristics of every module is calculated. At the end, the importance of genes in the computation module represents the association between genes and samples. The soft threshold for network construction was 5. Finally, we conducted in-depth research on the correlation between EMT and Wnt pathway genes.

### Consensus clustering and principal component analysis

Using the R software package "ConsensusClusterPlus" [10] from TCGA, based on the difference in expressing Wnt signaling pathway-associated genes in BLCA patients, we identified two subtypes (50 iterations, 80% resampling rate Pearson correlation, http://www.bioconductor.org/). We used the cumulative distribution function and consensus matrix to calculate the appropriate number of subtypes. Then, we drew Kaplan–Meier overall survival curves and the classification of clinical stages based on these two subtypes.

### Establishment and verification of risk scoring signature and line graph model

The LASSO-Cox algorithm [11] in the "glmnet" R software package was employed to identify the overall survival clinical outcome signature. We verified the prognostic prediction value of the signature conducted in TCGA–BLAD by using Sangerbox (http://sangerbox.com/). Sangerbox is an R-based comprehensive analysis tool for bioinformatics.

### Analysis of three essential genes in the immune microenvironment

As an algorithm, CIBERSORT [12] analyzes the ratio of cells in the body tissue gene expression matrix. As a genetic signature matrix, LM22 defines 22 immune cell subtypes that can be downloaded from the CIBERSORT website portal (https://cibersort.stanford.edu/). We used LM22 Matrix and cipher algorithm to analyze the ratio of CD8+ t cells. The result showed that the number of samples was P < 0.05. Subsequently, the "ESTIMATE" R software package [13] was employed to measure tumor unit (referring to the ratio of cancer cells in tumor), immune mark (referring to the infiltration of immune cells in tumor) and matrix mark (recording the presence of tumor cells).We evaluated the immune microenvironment panorama of the factors in signatures to calculate correlations among the Wnt pathway, the immune microenvironment, and immune responses.

### Cell source and transfection

The Cell Bank of the Chinese Academy of Sciences (China) provided BLCA cell lines (T24 and UM-UC-3). Both cell lines were grown at 37 degrees Celsius and 5% CO2. Among them, the culture of T24 cells was made in RPMI (Hyclone; GE Healthcare), and the culture of UM-UC3 cells was made in high glucose DMEM (Hyclone; GE Healthcare). Both media were supplemented with 10% fetal bovine serum (FBS; Biological Industries, Beit-HaEmek, Israel). We ordered small interfering RNA (siRNA) for PPP2CB from JTSBIO Co. (China). The sequence is below si-PPP2CB (sense: GGAACCAGGCUGCUAUCAUTT; anti-sense: AUGAUAGCAGCCUGGUUCCTT).

### Cell proliferation assay

We use BeyoClick™ EdU-488 Cell Proliferation Kit (Beyotime Biotechnology, China) to measure cell proliferation. We incubated the transfected cells according to the instructions and obtained images on a fluorescence microscope (Olympus Corporation, Japan), and used ImageJ software (National Institutes of Health, Bethesda, Maryland) Perform a cell count.

### Wound healing assay

We seeded the cells in a 6-well plate. After 48 h of transfection, when the cells reached confluence, we used a pipette tip to cut the cells to form a mechanical wound. Use an optical microscope to image at 0 and 24 h, and compare cell migration by calculating the size of the gap in each field of view.

### Cell migration assay

We put the 8 μm Transwell chamber into a 24-well plate (Corning Costar, Corning, New York, USA), and preliminarily added 600 μl of medium containing 10%-FBS to each bottom well, and inoculated each upper chamber at the same time 8000 cells in suspension in 200 μl FBS-free medium. After incubation at 5% CO2 and 37 °C for 24 h, the upper layer film was removed in the cells, while the cells migrated to the lower side is fixed in 4% paraformaldehyde, and stained with 1.0% crystal violet. Use a microscope to obtain an image.

### Immunoblotting assay

We extract total protein from the cells by RIPA lysis buffer with 1% PMSF, and then determine the protein concentration by the bicinchoninic acid assay (Beyotime Institute of Biotechnology). The protein samples isolated by PAGE are transferred to polyvinylidene fluoride membranes. Separate the membrane with anti-N-cadherin (1:5000, ab76011, Abcam, USA), anti-vimentin (1:1000, 5741S, CST, USA), anti-β-tubulin (1:1000, 2128S, CST, USA) and anti-PPP2CB (1:10000; ab168371; Abcam, USA) antibodies were incubated overnight at 4 °C. After that, the membrane and the secondary antibody were combined in a shaker at 37 °C for 1 h, and the image was captured applying the EasySee Western Blot kit (Beijing Transgenic Biotechnology Co., Ltd., Beijing, China) and the chemiluminescence system (Bio-Rad), California, USA).

### Statistics analysis

We applied the R packages "glmnet" and "survival" to perform multivariate, Kaplan–Meier analysis and LASSO-Cox regression to evaluate risk signature, and use the R package "survivalROC" to perform Roc curve analysis [14]. R software (version4.0.3; https://www.r-project.org/) was used to perform all statistical analyses. It was considered that P < 0.05 was greatly significant. GraphPad Prism was also applied for data analysis.

## Results

### The relationship between Wnt signaling pathway-related genes and phenotypic characteristics of BLCA

**Fig. 1:**
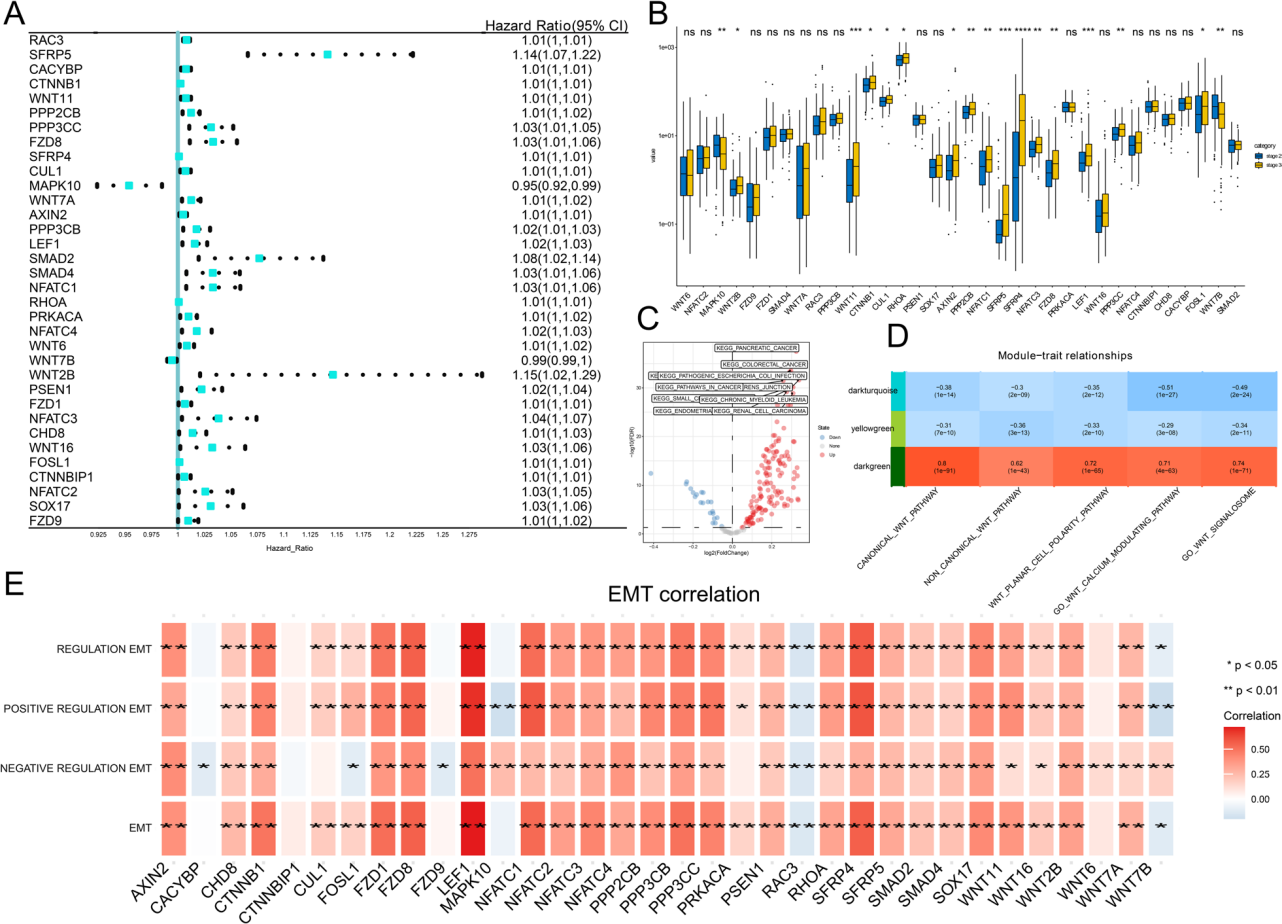
**A** Forest plot of hazard ratios, showing the prognostic value of Wnt-associated genes; > 1 indicates a poor prognostic factor, and < 1 represents a favorable prognostic factor. Univariate analysis showed that the higher the expression level, the worse the outcome. **B** In TCGA, the expression levels of 34 Wnt- associated genes in BLCA WHO II and WHO III (*P < 0.05, **P < 0.01, ****P < 0.0001, NSP ≥ 0.05). **C** The expression level of the Wnt pathway in various samples. **D** The association between the module identification and the sample. Module feature relationship. The module name is showed on the left. The number in the first row of the square is the association coefficient with the Wnt signal group, displayed at the top of every row, and the p-value is printed under the correlation in parentheses. These lines are colored on basis of the association between the module and the Wnt signaling pathway: dark green means positive correlation, dark turquoise, and yellow-green means negative correlation. **E** The correlation between 34 essential genes and EMT. The blue box represents a negative association, a red box represents a positive association, and a growth of color shows a growth of the association coefficient

On basis of the univariate Cox regression model, we found that 34 factors in the Wnt pathway were greatly related to OS. A forest map of risk scores displayed that 32 genes were risk elements and two genes (MAPK10 and WNT7B) were protective factors (Fig. 1A). The t-test of completely randomized data displayed the variation in the expression levels of these 34 genes in the clinical stages of BLCA and World Health Organization stages II and III (Fig. 1B). We used KEGG to analyze the metabolic pathways and signal transduction pathways of tumors significantly enriched in the Wnt pathway activation and inhibition groups. We found that these tumor-related pathways were also highly activated (Fig. 1C). Therefore, we believe that the Wnt signal pathway promotes cancer.

### The association between the Wnt signaling pathway and biological functions in BLCA

We used ssGSEA and WGCNA analysis to enrich the biological functions associated with the Wnt signaling pathway and tumors in TCGA. Three modules were obtained (dark turquoise, yellow-green and dark green) (Fig. 1D). Dark turquoise and yellow-green negatively correlated with Wnt pathways, and dark green positively correlated with Wnt pathways. In these three modules, we found that EMT-related biological GO terms were associated with the Wnt pathway (Table 1). EMT positively correlated with the activation of the Wnt signaling pathway. Therefore, we speculated that the high activation of Wnt signaling in BLCA would induce EMT in tumor cells, increasing tumor migration and invasion and affecting the outcome in terms of computational biology. Other biological functions were closely correlated with the activation or inhibition of Wnt signaling pathways, including angiogenesis, vascular endothelial cell migration, G protein-coupled receptor pathways, and the mitochondrial respiratory chain (Table 1).

**Table 1:** Correlation among WNT pathway and other GO functions

Correlation	GO_CANONICAL_WNT_SIGNALING_PATHWAY	GO_NON_CANONICAL_WNT_SIGNALING_PATHWAY	GO_REGULATION_OF_WNT_SIGNALING_PATHWAY_PLANAR_CELL_POLARITY_PATHWAY	GO_WNT_SIGNALING_PATHWAY_CALCIUM_MODULATING_PATHWAY	GO_WNT_SIGNALOSOME
GO_EPITHELIAL_TO_MESENCHYMAL_TRANSITION	0.79480331	0.583747	0.677845813	0.749249036	0.762904048
GO_G_PROTEIN_COUPLED_ACETYLCHOLINE_RECEPTOR_SIGNALING_PATHWAY	0.442942289	0.412743729	0.375230298	0.404088981	0.445448447
GO_NEGATIVE_REGULATION_OF_SPROUTING_ANGIOGENESIS	0.444334924	0.300323183	0.505019482	0.43604778	0.419748416
GO_NEGATIVE_REGULATION_OF_BLOOD_VESSEL_ENDOTHELIAL_CELL_MIGRATION	0.443234707	0.310783794	0.462866401	0.427546524	0.406001389
GO_G_PROTEIN_COUPLED_RECEPTOR_SIGNALING_PATHWAY_COUPLED_TO_CYCLIC_NUCLEOTIDE_SECOND_MESSENGER	0.631278967	0.545572181	0.703316308	0.522399396	0.540023623
GO_VASCULOGENESIS	0.650854042	0.426591528	0.555919121	0.637577912	0.644784758
GO_ACTIN_FILAMENT_BINDING	0.734403861	0.686136572	0.692503374	0.590242774	0.624590603
GO_MITOCHONDRIAL_RESPIRATORY_CHAIN_COMPLEX_III_ASSEMBLY	− 0.589167212	− 0.52442356	− 0.482988077	− 0.547371294	− 0.589942517
GO_RESPIRATORY_ELECTRON_TRANSPORT_CHAIN	− 0.50603574	− 0.470990736	− 0.403627026	− 0.556423377	− 0.540776029
GO_RESPIRATORY_CHAIN_COMPLEX_III	− 0.44836815	− 0.501114083	− 0.427534625	− 0.44733523	− 0.428247616
GO_MITOCHONDRIAL_ELECTRON_TRANSPORT_UBIQUINOL_TO_CYTOCHROME_C	− 0.425364356	− 0.46843121	− 0.418905826	− 0.441280101	− 0.403991375
GO_STRUCTURAL_CONSTITUENT_OF_RIBOSOME	− 0.432828749	− 0.40109856	− 0.334483352	− 0.452632376	− 0.491683488

According to these outcomes, the Wnt signaling pathway is closely related to EMT. We further explored the correlation between 34 Wnt pathway-related genes that affect BLCA outcome and EMT. We calculated the influence of these 34 genes on EMT (Fig. 1E) and found that PPP2CB, LEF1, CTNNB1 were positively correlated with EMT. RAC3 was significantly negatively related to EMT.

### The subgroup of Wnt signal expression in BLCA

**Fig. 2:**
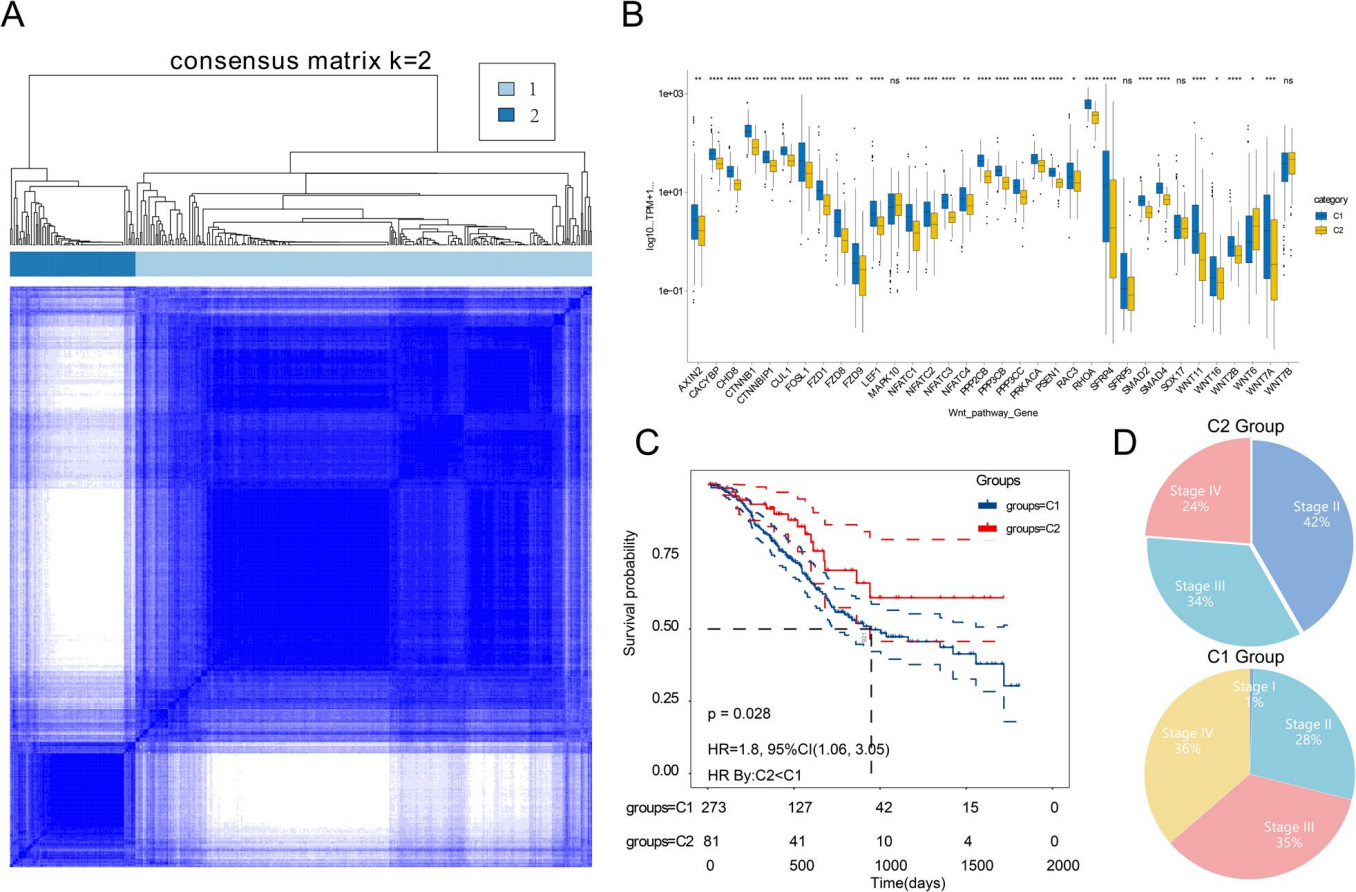
**A** The most suitable (k = 2) consensus clustering matrix. **B** Differences in the expression of 34 Wnt signaling pathway-associated genes in the two subtypes of BLCA in TCGA data set. **C** Kaplan–Meier overall survival curves of BLCA patients with two subtypes in TCGA data set. Significance: *P < 0.05, **P < 0.01, ***P < 0.001. **D** Clinical staging of two

Figure 1 illustrates univariate Cox analysis of 34 genes in the Wnt pathway that had significant regression coefficients and were associated with BLCA outcome and EMT. Based on these 34 genes, we classified the Wnt signaling pathway in patients with BLCA. The consensus clustering of Wnt-associated genes divides BLCA specimens into two clusters. Using the similarity with Wnt-related gene expression, we chose the value of k = 2 (Fig. 2A).

On basis of the expression of these 34 genes, the BLCA samples from TCGA data set were fallen into two subgroups (C1 and C2). We compared the expression differences of these genes in these subgroups (Fig. 2B). The high expression group was the C1 group, and the low expression group was the C2 group. The C1 group was the Wnt signaling pathway enrichment group, and the C2 group was the Wnt signaling pathway deficiency group. The C1 group had poor outcomes, and the C2 group had better outcomes. The risk ratio of the C1 group was 1.80 of the C2 group, and the confidence interval was 1.06–3.05 (P = 0.028) (Fig. 2C). According to the database, the proportions of clinical stages in the two groups differed. The proportion of stage II BLCA (WHO) in group C1 was 28%; the proportion of stage II BLCA (WHO) in group C2 was 42%; the proportion of stage IV BLCA (WHO) in group C1 was 36%, and the proportion of BLCA in group C2 Phase III (WHO) accounted for 24%. The difference between the two groups of Phase III (WHO) was small (Fig. 2D). These findings suggest that the BLCA outcome in Wnt signaling pathway enrichment group is worse. The outcome of stage IV patients was worse, the outcome of stage II patients was better, and no great variation was found between stage III patients and the Wnt signaling deficiency group.

### The prognostic prediction model associated with the Wnt pathway in BLCA

**Fig. 3:**
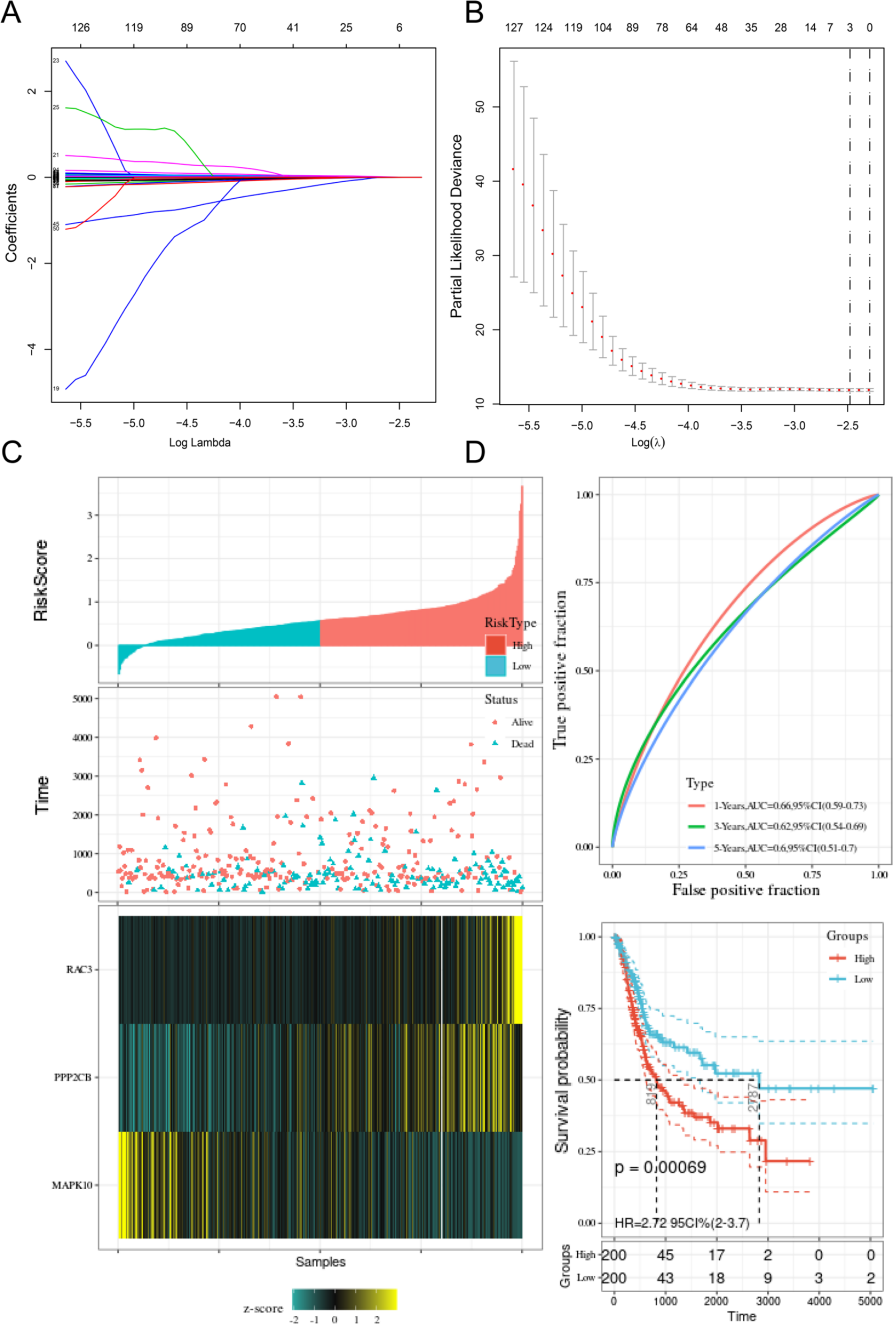
**A**, **B** LASSO-Cox regression shows independent prognostic genes. The upper picture displays the shrinkage of the coefficients, and the lower picture shows a tenfold cross-validation. **C** Risk mark signature on basis of three essential genes. The upper figure displays the calculation formula and the value of the risk mark; the middle figure displays the distribution of survival status on basis of the risk mark; the bottom figure displays the cluster heat map of the three essential genes. **D** ROC over time shows the accuracy of Nomogram in predicting 3-year overall survival and 5-year overall survival. According to the Kaplan–Meyer survival curve, the survival rates of BLCA patients with high-risk scores and low-risk scores are different

Having found that the Wnt signaling pathway was related to BLCA outcome, we tried to establish a set of prognostic risk scores associated with the Wnt pathway based on BLCA. We found 34 Wnt pathway-related genes. After applying the LASSO-Cox algorithm to these 34 genes, we identified MAPK10, RAC3, and PPP2CB as independent prognostic genes (Fig. 3A, B). Considering the role of these three genes in BLCA outcome, a risk mark signature was set up for the integration of the effects of these genes using LASSO-Cox regression coefficients (Fig. 3C). Risk score = − 0.001 * MAPK10 + 0.002 * RAC3 + 0.001 * PPP2CB.

Based on the median risk mark, BLCA patients were fallen into high-risk and low-risk groups in TCGA. The survival rate in the low-risk group was greatly higher than that of the high-risk group. According to the time-varying ROC curve, the region under the nomogram curve (AUC) used for the prediction of the 3-year survival rate was 0.62, while the 5-year survival rate predicted by the AUC was 0.6 (Fig. 3D).

**Fig. 4:**
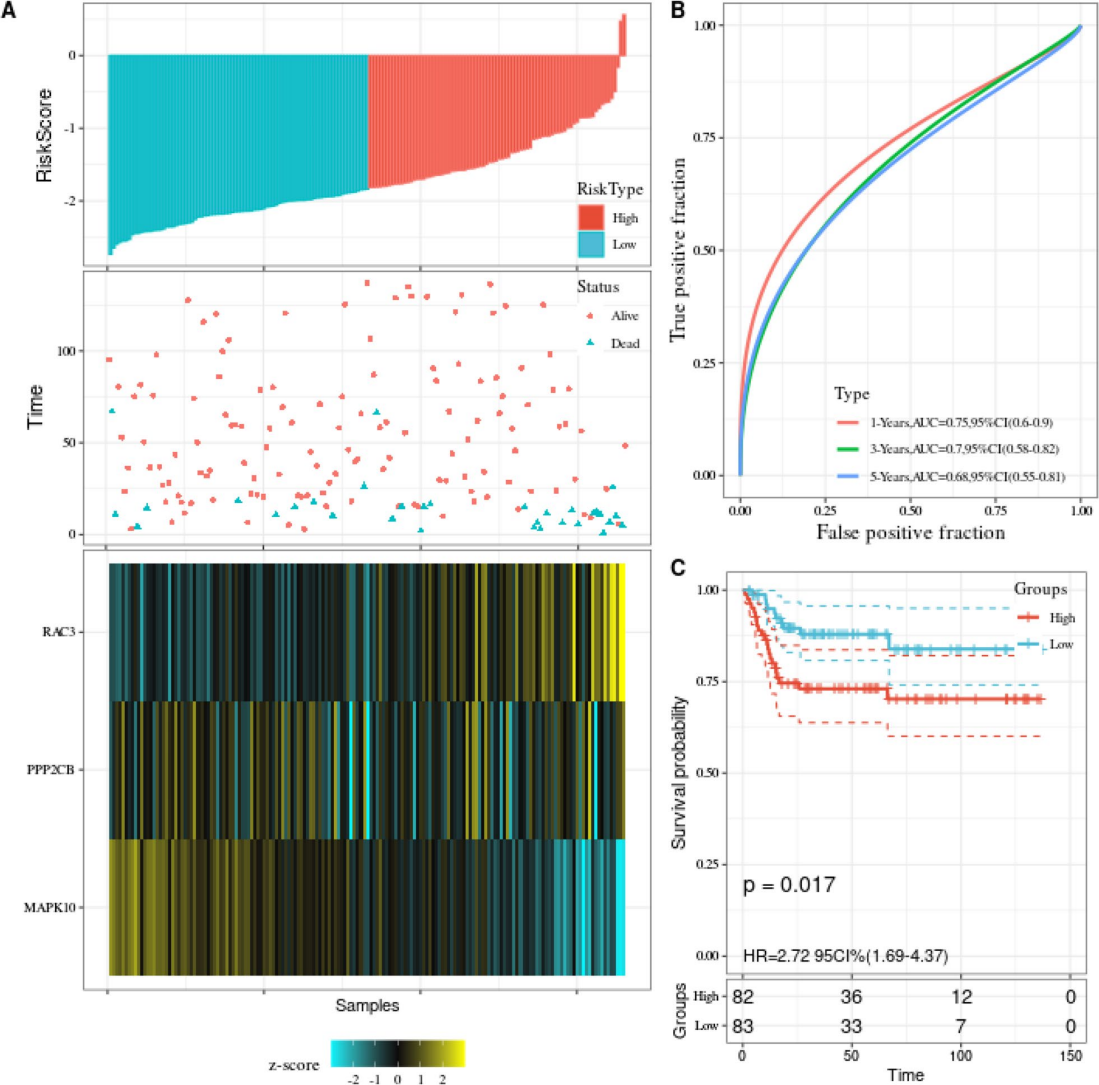
Confirm the prognostic value of the three Wnt-related genes in the GEO dataset. **A** Risk mark signature on basis of three basic genes. The upper figure displays the calculation formula and the value of the risk mark; the middle figure displays the distribution of survival status on basis of the risk mark; the bottom figure displays the cluster heat map of the three essential genes. **B** Time-dependent ROC displays the accuracy of the nomogram of 1-year overall survival prediction, 3-year overall survival prediction, and 5-year overall survival prediction. **C** Kaplan–Meyer survival curve displays the survival rate of BLCA patients with high-risk and low-risk marks

Universality is an essential indicator for evaluating prognostic models, and it is necessary to verify signatures externally using data from other sources. GEO data sets applying various chip platforms were selected for the confirmation of the prognostic predictive power of Wnt-associated genes and the signature. We found that these gene signatures had prognostic significance in GSE13507 (Fig. 4A–C).

### The association between the three genes and the immune microenvironment of BLCA

**Fig. 5:**
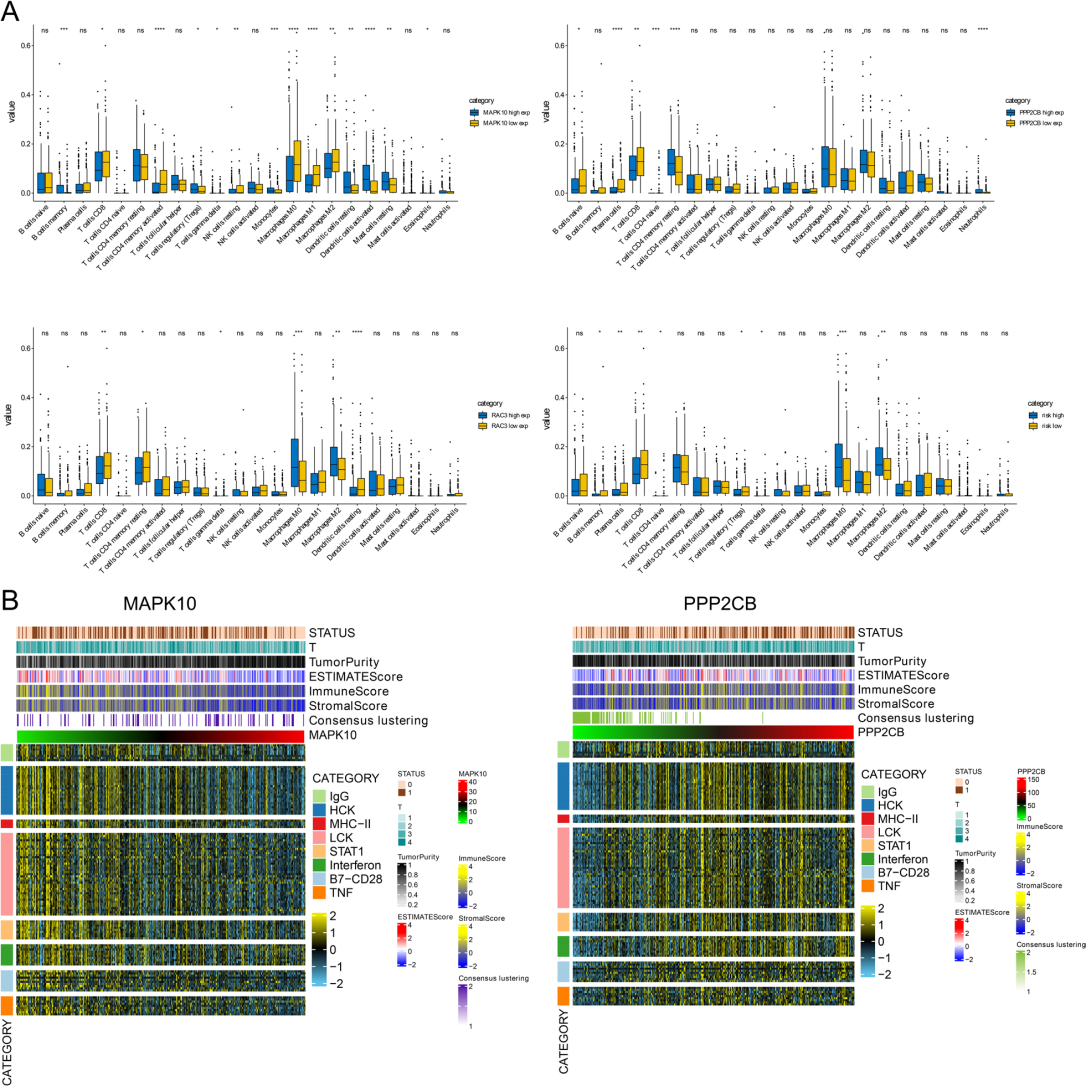
**A** Top left: The content of 22 immune cells in the high expression group and low expression group of MAPK10. Upper right: The content of 22 immune cells in the high expression group and low expression group of PPP2CB. Lower left: The content of 22 immune cells in the RAC3 high expression group and low expression group. Lower right: The content of 22 immune cells in the high-risk group and the low-risk group. **B** The image on the left shows the survival status of different expression levels of MAPK10, tumor stage, tumor purity, ESTIMATE score, immune score, matrix score, and immune response intensity (expression of eight immune-related genes). The image on the right displays the survival status of various expressions of PPP2CB, tumor stage, tumor purity, ESTIMATE mark, immune mark, matrix score, and the intensity of immune response (expression of eight immune-related genes). Subtypes of BLCA patients in TCGA

Various immune infiltration and anti-tumor effects in the immune tumor microenvironment are caused by immune cells and immune-related pathways. The immune infiltration status of the transcriptome of BLCA data was assessed by CIBERSORT algorithm. Eight immune-related genes in 22 immune cells were used to assess immune infiltration in BLCA. We analyzed the expression of MAPK10, RAC3, and PPP2CB in BLCA, the risk score, and the infiltration of immune cells (Fig. 5). By comparing with the MAPK10 high expression group, the MAPK10 low expression group had more M2 macrophages. Compared with the PPP2CB high expression group, the PPP2CB low expression group had fewer M2 macrophages (Fig. 5A).

The ESTIMATE algorithm was applied to assess the association between immune infiltration status and tumor purity, and the calculation of tumor stage, survival status, tumor purity, ESTIMATE mark, stroma mark, and the immune mark of TCGA was made (Fig. 5B). The heat map displayed that when the expression of MAPK10 is low, the purity of the tumor is lower, the immune score is higher, the stromal cell score is higher, and the expression of eight immune-associated genes is higher. When the expression of PPP2CB is low, the expression of the eight immune-related genes is low.

### Functional characteristics of PPP2CB

**Fig. 6:**
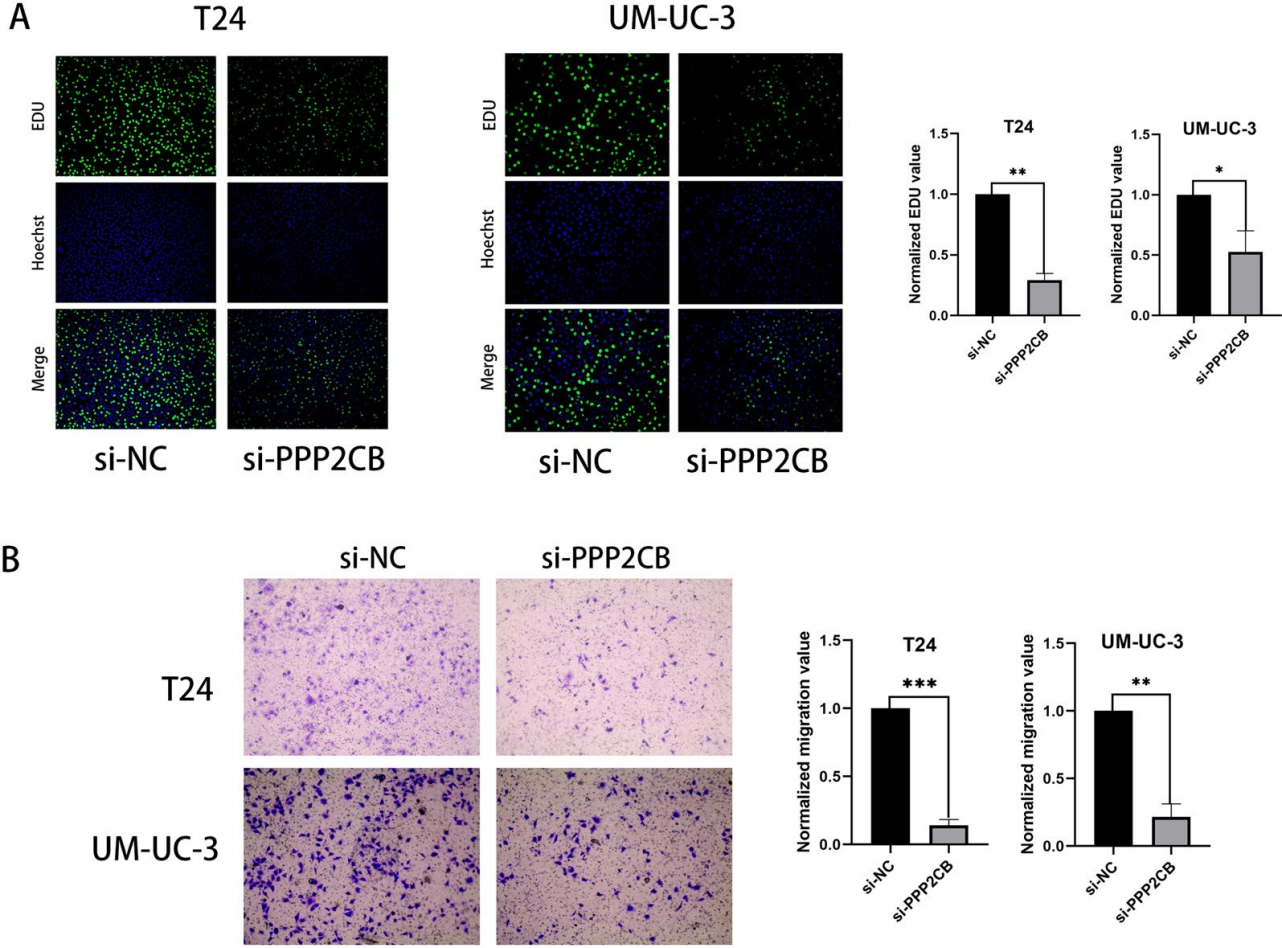
**A** The proliferation of T24 and UM-UC-3 after silencing the expressions of PPP2CB. **B** Migration of T24 and UM-UC-3 after silencing the expressions of PPP2CB. The analysis of data was made using the t-test, which was showed as the mean ± standard deviation

**Fig. 7:**
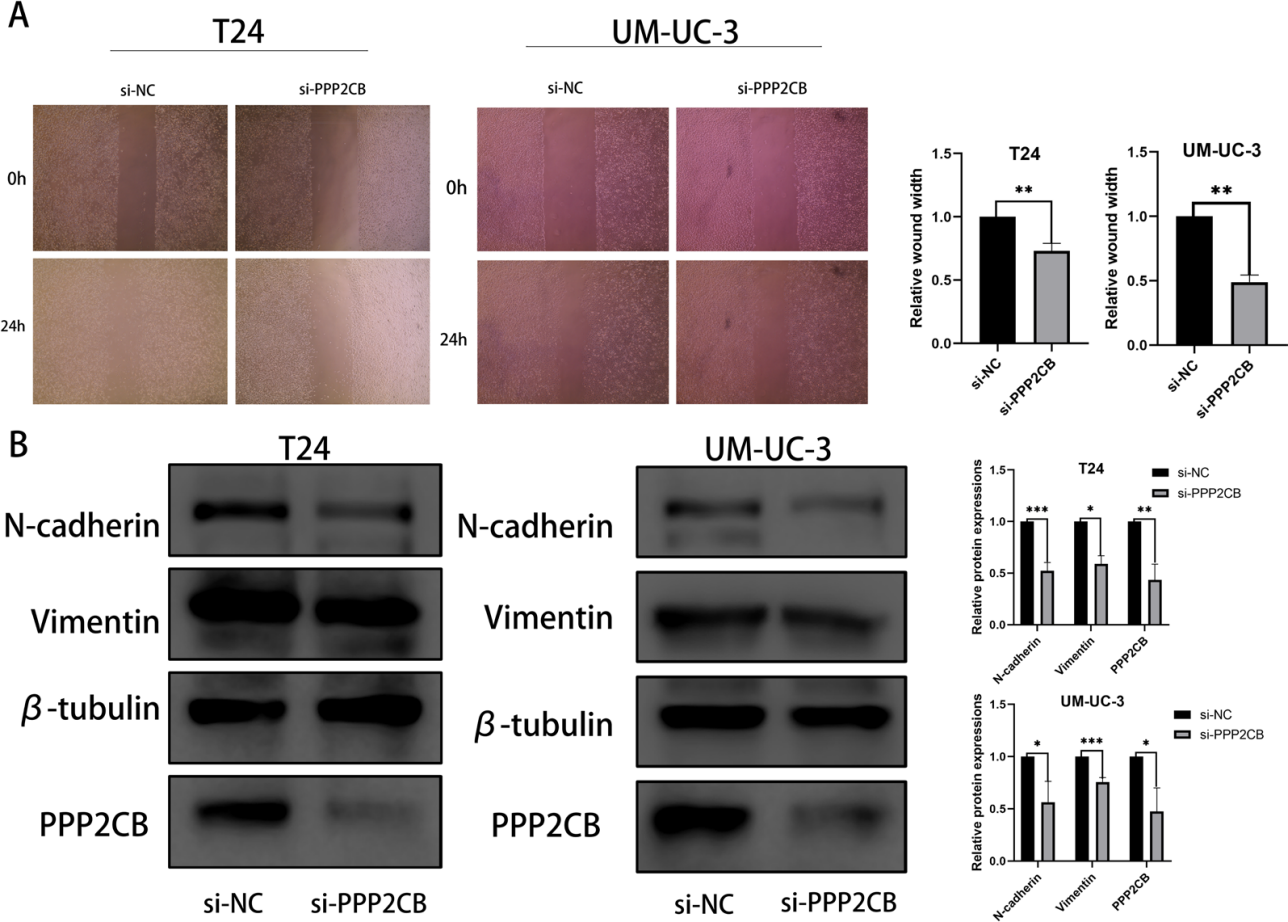
**A** Migration of T24 and UM-UC-3 after silencing the expressions of PPP2CB. The analysis of data was made by t-test, which was showed as the mean ± standard deviation. **B** The detection of PPP2CB, vimentin and N-cadherin proteins in T24 and UM-UC-3 was made after silencing the expressions of PPP2CB. *P < 0.05; **P < 0.01; ***P < 0.001

To further validate our bioinformatics analysis, we selected a risk gene (PPP2CB) and performed cell function experiments by silencing its expression using siRNA. To date, no studies have reported the function of this gene in BLCA cells. We performed functional verification to determine the possible effects of this gene on the BLCA cell lines T24 and UM-UC3. After downregulating the expression of PPP2CB, the proliferation and migration of BLCA cells were significantly inhibited (Figs. 6A, B; 7A). Using western blotting, it was observed that silencing PPP2CB led to downregulation of protein levels of N-cadherin and vimentin, suggesting that this gene promotes EMT in BLCA cells (Fig. 7B). These results provide us with a part of the experimental basis for verifying bioinformatics analysis and provide further insights for the further study of molecular functions and mechanisms.

## Discussion

Several biological phenomena were controlled by the Wnt signal transduction cascade throughout all animals' growth and adult life, and aberrant Wnt signaling emphasizes various pathologies in humans [15]. In this study, we found that Wnt signaling is related to the occurrence, growth, metastasis, and recurrence of BLCA, and the Wnt signaling pathway is a prognostic risk factor for BLCA.

In BLCA, many upstream and downstream molecular members of Wnt signaling participate in various biological functions. For example, Wnt signaling regulate angiogenesis [16–19], induces vascular endothelial cell migration [20, 21], and participates in G-protein-coupled receptor signal transmission [22–24], regulation of mitochondrial respiratory chain [25–28], and EMT.

Based on the WGCNA analysis, we found EMT-related GO terms strongly correlated with Wnt pathways. Therefore, we focused on the regulation mechanism of EMT and Wnt. EMT is a necessary process in morphogenesis. Epithelial cells lose cell-to-cell contact, polarity, and other epithelial cells' characteristics and obtain the unique characteristics of mesenchymal cells (including enhanced motility). The characteristics of EMT are the loss of E-cadherin on the plasma membrane, the increase of vimentin and fibronectin, and the growing accumulation of β-catenin in the nucleus. Except normal growth processes, EMT also participates in tumor development and progression. The initiation of tumor metastasis depends on the interaction between tumor cells and stromal cells, and EMT occurs in single cells. Hybrid EMT occurs in collectively migrating cells [29]. These findings suggest that EMT is closely related to tumor metastasis and recurrence.

EMT is regulated by signal transduction pathways including TGF-β, Notch, NF-κB, Wnt, and receptor tyrosine kinase [30]. The regulation of EMT by the Wnt signaling pathway has been determined including Wnt5a [31], FOXP3 [32], SERPINH1 [33], CUL4B [34], and SUFU [35], which can be used in BLCA, gastric cancer, and pancreatic cancer. Activating the Wnt signaling pathway induces EMT. The Wnt pathway also inhibits EMT, and EFEMP2 may inhibit BLCA progression through the Wnt/β-catenin pathway to prevent EMT [36]. The downregulation of E-cadherin causes the nuclear translocation of β-catenin and activation of canonical Wnt signaling [37]. As a marker gene of EMT, the gene *slug* also induces nuclear translocation of β-catenin [38]. Studies have shown a close association between the Wnt signaling pathway and EMT and two-way modulation between the two.

Because BLCA often recurs and has high invasiveness and metastasis, we explored the correlation between the Wnt signaling pathway and EMT and the impact of the Wnt pathway on BLCA outcome. We found that patients with highly activated Wnt signaling pathways had a more unsatisfactory outcome, suggesting that the Wnt signaling pathway is cancer-promoting. We also obtained five important prognostic independent risk factors for BLCA based on the Wnt pathway (MAPK10, PPP2CB, LEF1, CTNNB1, and RAC3). These Wnt signaling pathway genes are closely associated with EMT. PPP2CB, LEF1, CTNNB1 positively correlated with EMT; RAC3 significantly negatively correlated with EMT; MAPK10 positively regulated and inhibited EMT.

Wnt signal transduction and its crosstalk with various immune cells have both positive and negative effects on tumor progression. For example, it can renew white blood cells or it can promote immune tolerance and limit the anti-tumor response.

The interaction between inflammatory cells and cancer cells has been researched well. Signal pathways such as TGF-β, NF-κB and Notch have been confirmed to affect the occurrence of EMT. Changes in the tumor microenvironment are also closely related to tumor proliferation and metastasis, affecting the development of cancer and the prognosis of patients. Pro-inflammatory factors and immune-related factors also play different roles in the process of tumor metastasis. For example, mitochondrial reactive oxygen species (ROS) can further affect tumor metastasis by regulating the inflammatory response [39–41]. Thus some natural antioxidants or anti-inflammatory drugs may also be used to inhibit tumor metastasis [39, 42, 43]. In colorectal cancer, including the spectrum from normal colorectal adenoma to cancer, infiltrating macrophages display high levels of Wnt2 and Wnt5a. This finding suggests that the activation of paracrine Wnt by macrophages leads to cancer progression [44]. The current research showed that the Wnt/β-catenin pathway-related factors of the Wnt classic pathway andB cell transcription coactivator 9 (BCL9) were related to immune cell infiltration. The expression of BCL9/BCL9L negatively related to the infiltration of CD8+ T cells in triple negative breast cancer, and BCL9/BCL9L inhibited the infiltration of CD8+ T cells in the tumor microenvironment [45].

The Wnt pathway promotes tumor growth by inhibiting immune cell infiltration. Wnt ligands secreted by tumor cells stimulate tumor-related macrophages to polarize to the M2 subtype through the canonical Wnt signaling pathway, leading to tumor development and migration [46]. This finding was consistent with our results, showing that when PPP2CB is highly expressed, the infiltration of M2-type macrophages is more substantial. M2 type macrophages have lost the functions of tumor antigen peptide presentation and processing. When the number of M2 type macrophages increases, immunity weakens, and outcomes worsen, suggesting that PPP2CB is an oncogene. When the expression of MAPK10 is low, the infiltration of M2-type macrophages is more substantial, suggesting that MAPK10 is a tumor suppressor gene.

In summary, as the central transportation hub in tumor tissue, PPP2CB affects tumor cell migration by regulating EMT, and it also regulates the tumor immune microenvironment and affects tumor immunity by interacting with immune cells.

## Conclusion

We set up a three-gene signature on basis of Wnt signaling-related genes, EMT, and immune infiltration in BLCA. These genes affect tumor proliferation as members of the Wnt pathway family and affect tumor metastasis by regulating EMT, even participating in immune infiltration. We also identified PPP2CB as a risk factor for the activity, proliferation, and migration of BLCA cells and established relevant immune infiltration assessments to predict their survival rate accurately. We expect that our findings will deepen the understanding of the molecular mechanisms taking part in the occurrence and development of BLCA and provide a unique method for discovering predictive biomarkers and selecting targeted therapies.

## Supplementary Information

**Additional file 1. Table S1.** The list of WNT pathway genes name in KEGG.

## Abbreviations

CDF: Cumulative distribution function
ME: Module Eigengene
GS: Gene significance
GTEx: Genome Tissue Expression
ROC: Receiver Operating Characteristic
BLCA: Bladder cancer
WGCNA: Weighted correlation network analysis
EMT: Epithelial-mesenchymal transition
RNA-seq: RNA-sequencing
TCGA: The Cancer Genome Atlas
GEO: Gene Expression Omnibus
NMF: Non-Negative Matrix Factorization
ssGSEA: Single-sample gene set enrichment analysis
GSVA: Gene Set Variation Analysis
OS: Overall survival
BCL9: B cell transcription coactivator 9
ROS: Mitochondrial reactive oxygen species
